# Ozenoxacin suppresses sebum production by inhibiting mTORC1 activation in differentiated hamster sebocytes

**DOI:** 10.1111/1346-8138.17409

**Published:** 2024-08-01

**Authors:** Takamichi Kitano, Toshikazu Koiwai, Koki Fujikawa, Sachi Mori, Tatsumi Matsumoto, Takashi Sato

**Affiliations:** ^1^ Drug Development Research Laboratories, Kyoto R&D Center Maruho Co., Ltd. Kyoto Japan; ^2^ Department of Biochemistry, School of Pharmacy, School of Pharmacy Tokyo University of Pharmacy and Life Sciences Tokyo Japan; ^3^ Strategic Research Planning & Management Department, Maruho Shonan Innovation Lab Maruho Co., Ltd. Kanagawa Japan; ^4^ Global Business Development Department Maruho Co., Ltd. Osaka Japan

**Keywords:** acne vulgaris, anti‐bacterial agents, sebaceous glands, sebum, TOR serine–threonine kinases

## Abstract

Acne vulgaris is a complex condition involving factors that affect the pilosebaceous unit. A primary manifestation of acne pathology is the development of comedones, often linked to the overproduction of sebum resulting from 5α‐dihydrotestosterone (5α‐DHT) and insulin activity. Ozenoxacin is a topical quinolone that exhibits potent antibacterial activity against *Cutibacterium acnes* (*C. acnes*). It is commonly used to treat acne associated with this bacterium; however, its effect on sebum production within the sebaceous glands remains unclear. In this study, the effects of ozenoxacin on sebum production were examined using insulin‐ and 5α‐DHT‐differentiated hamster sebocytes. Ozenoxacin showed a dose‐dependent inhibition of lipid droplet formation and triacylglycerol (TG) production, which is a major component of sebum. In addition, it suppressed the expression of diacylglycerol acyltransferase 1, stearoyl‐CoA desaturase‐1, and perilipin‐1 mRNA, all important factors involved in sebum synthesis, in a dose‐dependent manner. Moreover, ozenoxacin decreased phosphorylated 40S ribosomal protein S6 levels downstream of the mechanistic/mammalian target of rapamycin complex 1 (mTORC1), without altering the phosphorylation of Akt, an upstream regulator of mTORC1, in both insulin‐ and 5α‐DHT‐treated hamster sebocytes. Interestingly, nadifloxacin, but not clindamycin, exhibited a similar suppression of sebum production, albeit with lesser potency compared with ozenoxacin. Furthermore, a topical application of a 2% ozenoxacin‐containing lotion to the auricle skin of hamsters did not affect the size of the sebaceous glands or epidermal thickness. Notably, it decreased the amount of TG on the skin surface. The results provide novel insights into the sebum‐inhibitory properties of ozenoxacin, indicating its potential efficacy in controlling microbial growth and regulating sebum production for acne management.

## 1 INTRODUCTION

Acne vulgaris is one of the most common skin disorders in adolescents and young adults, affecting up to 80% of the population at some point in their lives.^1^ It is a multifactorial condition of the pilosebaceous unit, resulting from the interplay of several factors, including excessive sebum secretion from sebaceous glands, hyperkeratosis of hair follicles, colonization of *Cutibacterium acnes* (*C. acnes*) in hair follicles, and inflammatory reactions triggered by cytokines, such as tumor necrosis factor α and interleukin 6.^2^

The initial manifestation of acne is the formation of comedones, which serve as precursors to papules and pustules.^2^ They are associated with the overproduction of sebum by androgens, such as testosterone and 5α‐dihydrotestosterone (5α‐DHT), particularly in adolescents.^3^, ^4^ Sebum production is induced by insulin, insulin‐like growth factor 1 (IGF‐1), and peroxisome proliferator‐activated receptors, such as prostaglandin J_2_.^5^, ^6^, ^7^ Thus, managing inflammatory acne and the formation of comedones associated with heightened sebum production is important.

Benzoyl peroxide, adapalene, and antibacterial agents, such as nadifloxacin and clindamycin, are recommended topical treatments for acne vulgaris in Japan.^8^ Adapalene has been reported to inhibit epidermal keratinocyte differentiation,^9^ whereas nadifloxacin and clindamycin exert antibacterial effects against *C. acnes*.^10^ Benzoyl peroxide exhibits antibacterial activity against *C. acnes* through free radical generation and exfoliates the stratum corneum.^11^, ^12^ Conversely, Gloor et al.^13^ reported that the repeated application of 10% benzoyl peroxide to the auricle of hamsters reduces the area fraction of sebaceous glands, as determined by [^3^H]‐thymidine‐labeled sebaceous gland cells and the abundance of mid‐mitotic sebaceous gland cells. In addition, Sato et al.^14^, ^15^ demonstrated that adapalene, nadifloxacin, and clindamycin inhibit sebum production in insulin‐stimulated hamster sebocytes. Taken together, these well‐known antiacne agents exhibit novel pharmacological actions that may affect the activity of sebaceous glands.

Ozenoxacin is a topical quinolone known for its potent antibacterial activity against a broad spectrum of bacteria.^16^, ^17^, ^18^, ^19^, ^20^, ^21^ Kawashima et al.^22^ recently demonstrated that it has excellent therapeutic efficacy in a 2% lotion formulation against inflammatory acne in patients with acne vulgaris. Consequently, the Japanese guidelines for treating acne vulgaris in 2023 recommend using ozenoxacin‐containing medications for inflammatory in addition to topical antibacterial agents such as nadifloxacin and clindamycin.^8^ However, there is limited information describing the underlying therapeutic mechanisms of ozenoxacin for acne vulgaris. Although nadifloxacin and clindamycin reduce sebum production and inflammatory reactions in hamster sebocytes,^15^ it is unclear whether ozenoxacin affects sebaceous gland function. In this study, we determined the effect of ozenoxacin on sebum production in hamster sebaceous glands in vitro and in vivo.

## 2 METHODS

### 2.1 Cell culture and treatments

Hamster sebocytes (1 × 10^4^ cells per cm^2^) were cultured in a 12‐well culture plate using Dulbecco's modified Eagle's medium/Ham's F12 (DMEM/F12) (1:1) (Invitrogen, Carlsbad, CA, USA) supplemented with 6% heat‐inactivated fetal bovine serum (FBS) (JRH Bioscience, Tokyo, Japan), 2% human serum, (ICN Biochemicals, Costa Mesa, CA, USA), and 0.68 mM L‐glutamine (Invitrogen).^23^ Two days after plating, the cells were treated every 3 days for up to 9 days with or without ozenoxacin (Fujifilm Toyama Chemical, Tokyo, Japan), nadifloxacin (Tokyo Chemical Industry, Tokyo, Japan), or clindamycin (Sigma‐Aldrich, Missouri, USA) in the presence or absence of insulin (10 nmol/L) (Sigma‐Aldrich) or 5α‐DHT (10 μmol/L) (Sigma‐Aldrich). The concentration of the antibiotics ranged from 3 to 100 μmol/L. Ozenoxacin and nadifloxacin, which have low solubility in water, but high solubility in NaOH, were administered in a medium supplemented with the equivalent NaOH as a negative control. Clindamycin, which is highly water‐soluble, was dissolved in water and diluted to each concentration with medium. Throughout these experiments, hamster sebocytes were used up to the third passage.

### 2.2 Oil red O staining

The sebocytes were rinsed with Dulbecco's phosphate‐buffered saline (PBS) (Sigma‐Aldrich) and fixed for 1 h with 4% paraformaldehyde phosphate buffer solution (Fujifilm Wako Pure Chemical, Tokyo, Japan) at 20–25°C. The cells were stained for 10 min with 0.3% Oil Red O (Waldeck GmbH & Co. KG, Münster, Germany) dissolved in isopropanol: distilled H_2_O (3:2, v/v) at 37°C, followed by a 5‐min treatment with Mayer's hematoxylin solution (Sakura Finetek Japan, Osaka, Japan) at 20–25°C. Subsequently, the stained cells were rinsed with PBS and examined using an inverted microscope equipped with a microscope digital camera (Olympus Corp., Tokyo, Japan). The quantification of the area stained with Oil Red O was performed using the free software “Fiji‐ImageJ” and a defined set of intensity thresholds was applied to all images.

### 2.3 TG measurement

Sebocytes were collected using a 0.25% trypsin/1 mmol/L EDTA solution and subjected to triacylglycerol (TG) quantitation using Aqua‐auto Kainos TG‐II (Kainos Laboratories, Tokyo, Japan) based on previously described methods.^23^ Intracellular TG levels were determined using an authentic trioleinate‐standard solution (0.6 mg/mL). In addition, intracellular DNA content was measured using salmon sperm DNA (6.25–100 μg/mL) (Fujifilm Wako Pure Chemical) and 3,5‐diaminobenzoic acid dihydrochloride (400 mg/mL) (Sigma‐Aldrich) as described previously.^23^

### 2.4 Analysis of sebum production by Nile red and Calcein‐AM staining

Hamster sebocytes in a 96‐well plate were treated every 2 days for 7 days with KU‐0063794 (0.1, 0.5, and 1 μmol/L) and LY294002 (5, 10, and 20 μmol/L) in the presence or absence of 10 nmol/L insulin and 10 μmol/L 5α‐DHT. The cells were stained with Nile Red (1 mg/mL) (Sigma Chemical) at 37°C for 30 min. The fluorescent intensity of the Nile Red‐stained cells was measured by a multimode microplate reader SpectraMax iD3 (Molecular Devices, San Jose, CA, USA) at 485 nm (excitation) and 565 nm (emission). The cells were stained with Calcein‐AM (0.5 mg/mL) (Dojindo Laboratories, Kumamoto, Japan) at 37°C for 30 min. The fluorescence intensity of the Calcein‐AM‐stained cells was measured with a multimode microplate reader SpectraMax iD3 (Molecular Devices) at 475 nm (excitation) and 515 nm (emission). Relative sebum production was quantified by the ratio of the fluorescent intensity of the Nile Red per Calcein‐AM, expressed as the mean value of the control at 100%.

### 2.5 Real‐time polymerase chain reaction

Total RNA was extracted from sebocytes using the RNeasy Plus Mini Kit (Qiagen, Hilden, Germany). Subsequently, a portion of the RNA (500 ng) was reverse‐transcribed into cDNA using the high‐capacity cDNA reverse transcription kit (Thermo Fisher Scientific, Waltham, MA, USA). DNA amplification was done using PowerSYBR® Green PCR Master Mix (2×) (Takara Bio, Shiga, Japan) and specific primers for hamster diacylglycerol acyltransferase 1 (DGAT‐1), perilipin‐1, stearoyl‐CoA desaturase‐1 (SCD‐1), and glyceraldehyde‐3‐phosphate dehydrogenase (GAPDH). The primer sequences were as follows: DGAT‐1: 5'‐TCAAGTGGGGCTGATCCAAC‐3' (sense) and 5'‐AGCTTCAAGAGCCGCTCAAT‐3' (antisense); perilipin‐1: 5'‐GATCCCAGCCCTTCAATACCC‐3' (sense) and 5'‐GATGCTGTTCCTGGCG‐3' (antisense); (SCD‐1): 5'‐CGAGAGAATATCCTGGTTTCCC‐3' (sense) and 5'‐TCATAGGGGAAGGCGTGGT‐3' (antisense); (GAPDH): 5'‐CAGAACATCATCCCTGCAT‐3' (sense) and 5'‐TAGGAACACGGAAGGCCAT‐3' (antisense). The results were normalized to GAPDH and analyzed using QuantStudio 7 Flex Real‐Time PCR and the StepOnePlus system (Thermo Fisher Scientific).

### 2.6 Phosphorylation of mTOR signaling‐related proteins

Phosphorylation of the mechanistic/mammalian target of rapamycin complex 1 (mTOR‐1) signaling‐related proteins, 40S ribosomal protein S6 (S6RP), and Akt was determined using the Phospho‐S6RP (Ser235/236), Phospho‐AKT1/2/3 (Ser473), and alpha‐tubulin housekeeping (PerkinElmer, Waltham, MA, USA) cellular assay kits. Cell lysates were prepared using a lysis buffer provided with the kit, and an aliquot of the lysate was used for the measurement of phosphorylated S6RP (p‐S6RP), phosphorylated Akt (p‐Akt), and α‐tubulin, following the manufacturer's instructions.

### 2.7 Animals

Five‐week‐old female Syrian hamsters (Japan SLC, Shizuoka, Japan) were provided access to water and standard laboratory food ad libitum. They were housed under controlled environmental conditions, with a temperature of 23 ± 3°C, a relative humidity of 50% ± 20%, and a 12‐h light:dark cycle, with lights on from 7:00 to 19:00 daily. The Laboratory Animal Committee of Maruho Co., Ltd obtained ethical approval for all experimental procedures.

### 2.8 In vivo oil red O and hematoxylin and staining

A lotion containing 2% ozenoxacin (Zebiax lotion®, Maruho, Osaka, Japan) or placebo (the base formulation of lotion containing 2% ozenoxacin) was applied topically to both sides of the right auricle skin of hamsters once a day for 14 days. The day after the final application, the right auricle was harvested and immersed for at least 4 h in 4% paraformaldehyde phosphate buffer solution at 4°C for fixation. Sucrose replacement was done by immersing the auricle in 7.5% sucrose solution for 5 min three times, followed by overnight immersion in 15% sucrose solution, and in 20% sucrose solution, both during refrigeration. The auricular tissues were embedded in OCT compound, frozen in isopentane, and cooled with liquid nitrogen. Frozen tissue sections were prepared using a cryostat, and the resulting sections (8 μm) were obtained. The sections were rinsed with distilled water and 60% isopropanol, followed by staining with 0.3% Oil Red O in isopropanol: distilled H_2_O (3:2, v/v) for 10 min at 37°C. After staining, the sections were rewashed with distilled water and 60% isopropanol, followed by staining with Mayer's hematoxylin solution for 5 min at 20°C to 25°C. Three views per section were examined using an inverted microscope equipped with a digital camera for microscopy (Olympus Corp.). Epidermal thickness and sebaceous gland area were measured using data capture software (cellSens Standard Ver. 1.12; Olympus).

### 2.9 Measurement of TG on the auricle skin surface of hamsters

The right auricle skin of the hamsters, which was topically treated with the 2% ozenoxacin lotion or placebo as described previously, was wiped with acetone‐soaked cotton. One hour after wiping, sebum on the skin surface was extracted with 3 mL of acetone for 1 min, repeated three times using glass test tubes. TG levels in the extract were analyzed using automatic thin‐layer chromatography, Iatroscan (Iatron Laboratories, Tokyo, Japan).

### 2.10 Statistical analysis

In the study using hamster sebocytes, the statistical significance of differences between the means of the control and treated groups was assessed using Tukey's multiple comparison test, Student's *t‐*test. In the study using hamster auricles, the statistical significance of differences between the means of treatment groups were analyzed by one‐way analysis of variance (one‐way ANOVA), followed by post hoc Fisher's least significant difference test. Mean differences were considered statistically significant at *P* < 0.05. The analyses were done using EXSUS statistics software (ver. 8.1.0; EP Croit, Tokyo, Japan).

## 3 RESULTS

### 3.1 Effects of ozenoxacin on insulin‐induced sebaceous lipogenesis induced in hamster sebocytes

**FIGURE 1:**
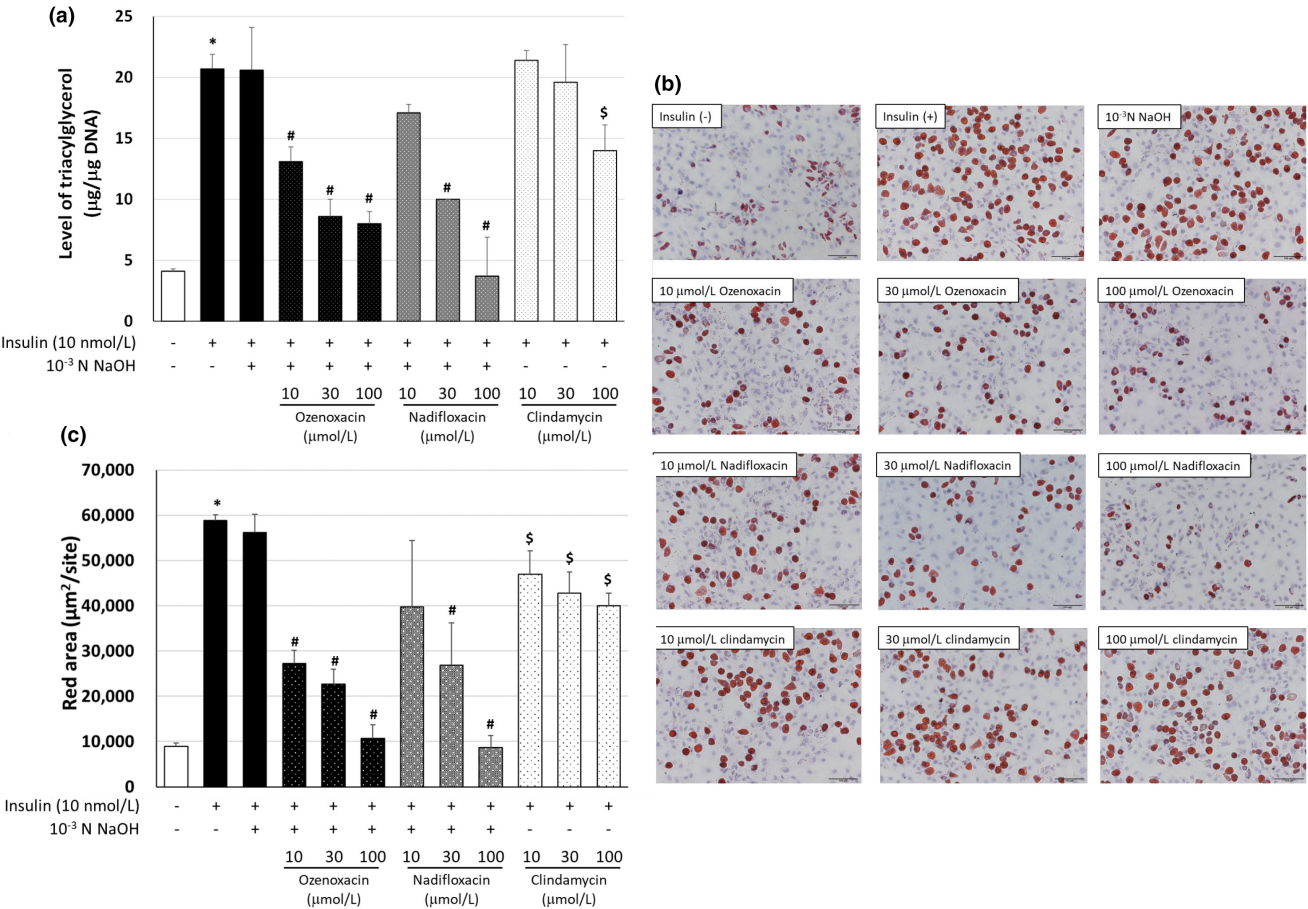
Effect of antimicrobial agents on triacylglycerol (TG) production and sebum accumulation in insulin‐differentiated hamster sebocytes. Hamster sebocytes were treated every 3 days for 9 days with ozenoxacin (10, 30, and 100 μmol/L), nadifloxacin (10, 30, and 100 μmol/L), and 10^−3^ N NaOH‐free clindamycin (10, 30, and 100 μmol/L) in the presence or absence of 10 nmol/L insulin. (a) The cells were homogenized, and intracellular TG and DNA were measured. Data are presented as means ± standard deviation of quadruplicate wells. (b) The cells were stained with Oil Red O. Scale bars = 100 μm. (c) The area stained with Oil Red O was quantified. *P < 0.05 compared with the untreated group (Aspin‐Welch's *t*‐test). ^#^ *P* < 0.05 compared with insulin +10^−3^ N NaOH (Tukey's multiple comparison test). ^$^ *P* < 0.05 compared with insulin (Tukey's multiple comparisons test).

Both insulin and androgens play a role in the pathogenesis of acne vulgaris.^3^, ^4^, ^6^, ^24^ Sebocytes accumulate lipid droplets intracellularly, resulting from an increase in sebum, which mainly consists of TG,^23^ therefore we determined the effects of ozenoxacin on lipid droplet formation and TG production in hamster sebocytes differentiated with insulin, and compared them with the effects of nadifloxacin and clindamycin. As shown in Figure 1a, ozenoxacin suppressed insulin‐induced TG production in hamster sebocytes in a dose‐dependent manner (with a maximum inhibition of 76.5% at 100 μmol/L). In addition, ozenoxacin decreased insulin‐enhanced intracellular lipid droplet formation in a dose‐dependent manner (Figure 1b,c). A similar suppression of TG production and lipid droplet formation by nadifloxacin and clindamycin was observed in insulin‐treated sebocytes (with a maximum inhibition of 102.5% and 40.6% at 100 μmol/L, respectively) (Figure 1a–c). Thus, similar to nadifloxacin and clindamycin,^15^ the results indicate that ozenoxacin inhibits the production and intracellular accumulation of TG in hamster sebocytes.

### 3.2 Effects of ozenoxacin on 5α‐DHT‐induced sebaceous lipogenesis in hamster sebocytes

**FIGURE 2:**
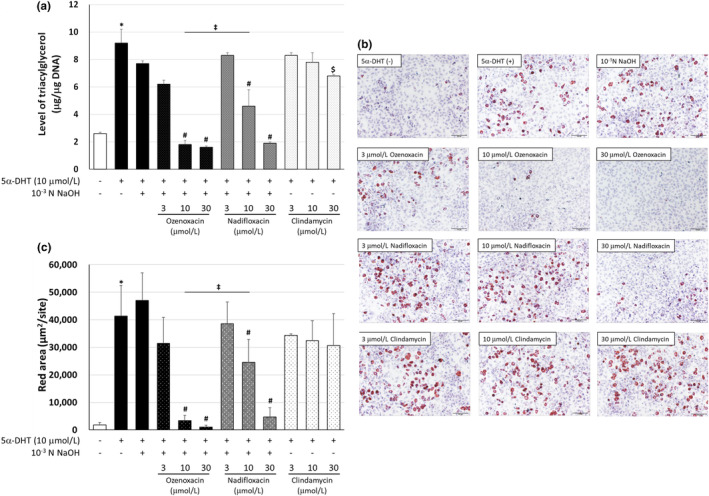
Effect of antimicrobial agents on triacylglycerol (TG) production and sebum accumulation in 5α‐dihydrotestosterone (5α‐DHT)‐differentiated hamster sebocytes. Hamster sebocytes were treated every 3 days for 9 days with ozenoxacin (10, 30, and 100 μmol/L), nadifloxacin (10, 30, and 100 μmol/L), and 10^−3^ N NaOH‐free clindamycin (10, 30, and 100 μmol/L) in the presence or absence of 10 μmol/L 5α‐DHT. (a) The cells were homogenized, and intracellular TG and DNA were measured. Data are presented as means ± standard deviation of quadruplicate wells. (b) The cells were stained with Oil Red O. Scale bars = 100 μm. (c) The area stained with Oil Red O was quantified. *P < 0.05 compared with the non‐treated group (Aspin‐Welch's *t*‐test). ^#^ *P* < 0.05 compared with 5α‐DHT + 10^−3^ N NaOH (Tukey's multiple comparison test). ^‡^ *P* < 0.05 compared with 5α‐DHT + ozenoxacin 10 μmol/L (Tukey's multiple comparison test). ^$^ *P* < 0.05 compared with insulin (Tukey's multiple comparisons test).

When hamster sebocytes were treated with ozenoxacin (3–100 μmol/L) in the presence of 5α‐DHT, ozenoxacin inhibited 5α‐DHT‐augmented TG production in a dose‐dependent manner (with a maximum inhibition of 119.4% at 30 μmol/L) (Supporting Information Figure S1). Based on this finding, the concentration range for ozenoxacin (3–30 μmol/L) was fixed, and the effects of ozenoxacin on TG production and intracellular accumulation were determined in hamster sebocytes. Ozenoxacin suppressed 5α‐DHT‐induced TG production (with a maximum inhibition of 115.6% at 10 μmol/L), accompanied by a reduction in intracellular lipid droplet formation in hamster sebocytes in a dose‐dependent manner (Figure 2). Similar inhibitory effects were observed following nadifloxacin treatment (114.3% inhibition at 30 μmol/L), whereas clindamycin had minimal effect (36.4% at 30 μmol/L). None of the antimicrobials used in this study exhibited toxicity to hamster sebocytes under the experimental conditions (Table 1), therefore the results indicate that ozenoxacin inhibits 5α‐DHT‐augmented TG production and intracellular lipid droplet formation in hamster sebocytes, similar to nadifloxacin.

**TABLE 1:** No cytotoxicity of ozenoxacin, nadifloxacin, or clindamycin in insulin‐ or 5α‐dihydrotestosterone (5α‐DHT)‐treated hamster sebocytes.

[A]		
Compound (μmol/L)		Cytotoxicity (%)
Ozenoxacin	10	0.0
	30	0.0
	100	0.0
Nadifloxacin	10	0.0
	30	0.0
	100	1.2
Clindamycin	10	0.1
	30	0.0
	100	1.3

[B]		
Compound (μmol/L)		Cytotoxicity (%)
Ozenoxacin	3	0.0
	10	0.0
	30	0.0
Nadifloxacin	3	0.0
	10	0.0
	30	0.0
Clindamycin	3	0.0
	10	0.0
	30	0.0

*Note*: Hamster sebocytes were treated with ozenoxacin, nadifloxacin, and clindamycin in the presence or absence of insulin (a, b) and 5α‐DHT (c, d), as depicted in Figures 1 and 2. Lactate dehydrogenase activity was measured in the cell supernatants using the Cytotoxicity Detection Kit Plus (Roche) according to the manufacturer's instructions. Cytotoxicity was calculated using 2% Triton X‐100 as a positive control, and no cytotoxicity was considered when the value was 10% or less.

### 3.3 Suppression of DGAT‐1, SCD‐1, and perilipin‐1 gene expression by ozenoxacin in insulin‐ and 5α‐DHT‐differentiated hamster sebocytes

**FIGURE 3:**
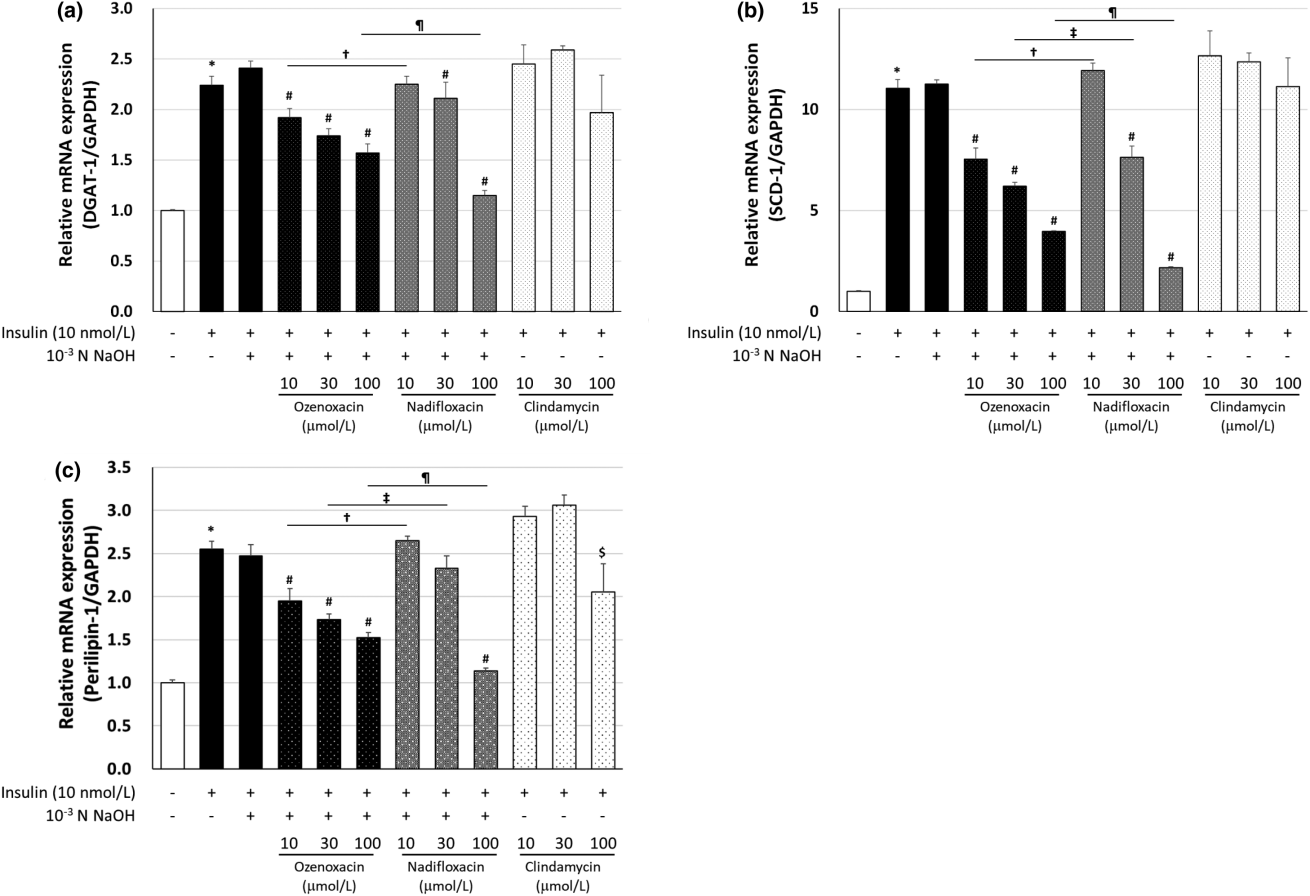
Regulation of *DGAT‐1, SCD‐1*, and *perilipin‐1* mRNA expression in insulin‐differentiated hamster sebocytes. Hamster sebocytes were treated with ozenoxacin, nadifloxacin, and clindamycin in the presence or absence of insulin, as shown in Figure 1. Total RNA was extracted from cells and the relative expression of *DGAT‐1* (a), *SCD‐1* (b), and *perilipin‐1* (c) was measured by real‐time PCR. Data are presented as means ± standard deviation of quadruplicate wells. *P < 0.05 compared with the non‐treated group (Student or Aspin‐Welch's *t*‐test). ^#^ *P* < 0.05 compared with insulin +10^−3^ N NaOH (Tukey's multiple comparison test). ^†^ *P* < 0.05 compared with insulin + ozenoxacin 10 μmol/L (Tukey's multiple comparison test). ^‡^ *P* < 0.05 compared with insulin + ozenoxacin 30 μmol/L (Tukey's multiple comparison test). ^¶^ *P* < 0.05 compared with insulin + ozenoxacin 100 μmol/L (Tukey's multiple comparison test). ^$^ *P* < 0.05 compared with insulin (Tukey's multiple comparison test).

**FIGURE 4:**
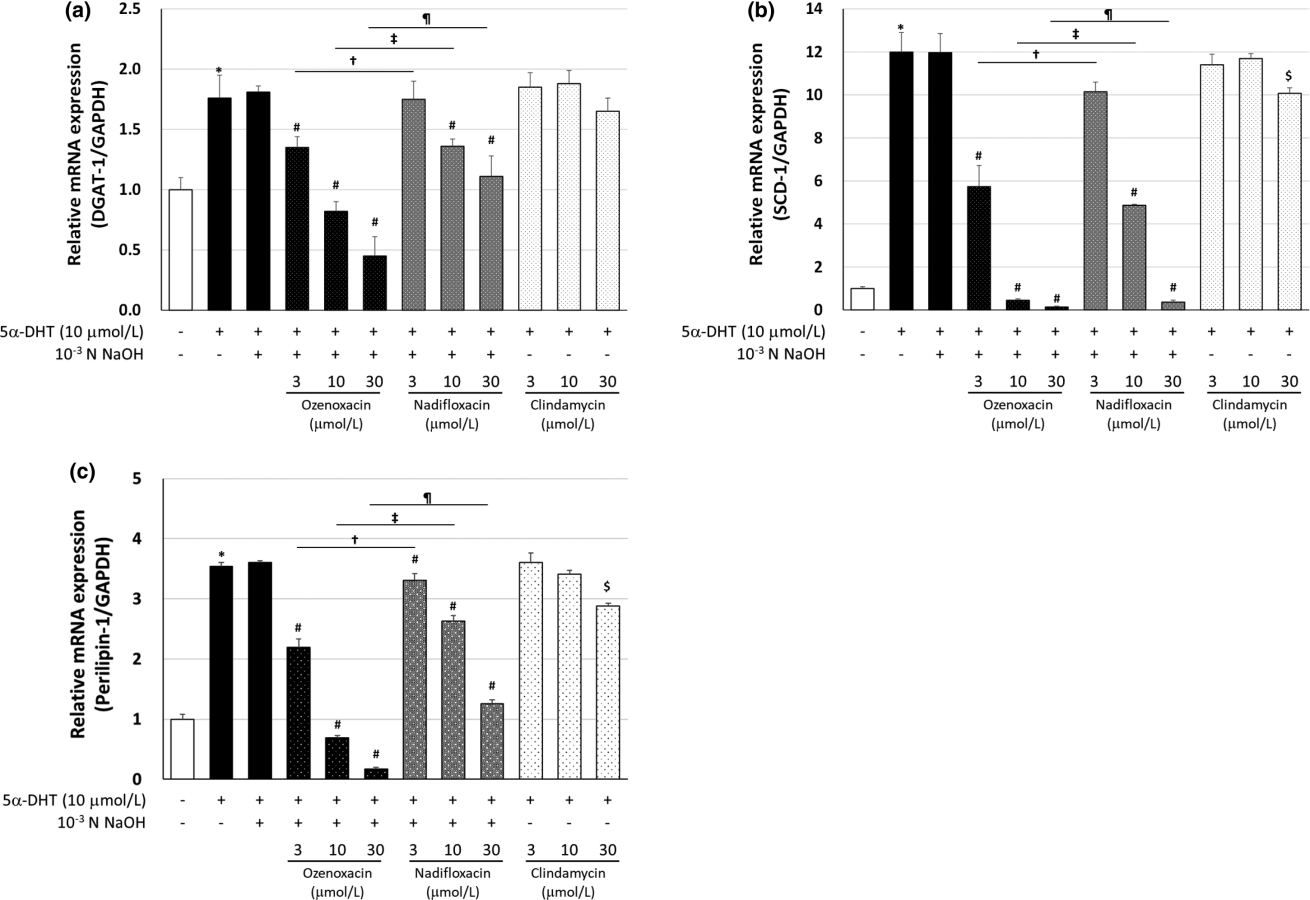
Regulation of *DGAT‐1, SCD‐1*, and *perilipin‐1* mRNA expression in 5α‐dihydrotestosterone (5α‐DHT)‐differentiated hamster sebocytes. Hamster sebocytes were treated with ozenoxacin, nadifloxacin, and clindamycin in the presence or absence of 5α‐DHT, as shown in Figure 2. Total RNA was extracted from cells and relative expression of *DGAT‐1* (a), *SCD‐1* (b), and *perilipin‐1* (c) was measured by real‐time PCR. Data are presented as means ± standard deviation of quadruplicate wells. *P < 0.05 compared with the non‐treated group (Student or Aspin‐Welch's *t*‐test). ^#^ *P* < 0.05 compared with insulin +10^−3^ N NaOH (Dunnett's multiple comparison test). ^†^ *P* < 0.05 compared with 5α‐DHT + ozenoxacin 3 μmol/L (Tukey's multiple comparison test). ^‡^ *P* < 0.05 compared with 5α‐DHT + ozenoxacin 10 μmol/L (Tukey's multiple comparison test). ^¶^ *P* < 0.05 compared with 5α‐DHT + ozenoxacin 30 μmol/L (Tukey's multiple comparison test). ^$^ *P* < 0.05 compared with insulin (Dunnett's multiple comparison test).

To elucidate the molecular mechanisms underlying ozenoxacin‐mediated inhibition of TG production and sebum accumulation, we examined the effects of various antimicrobial agents on the expression of the *DGAT‐1*, *SCD‐1*, and *perilipin‐1* genes in insulin‐and 5α‐DHT‐differentiated hamster sebocytes. These genes regulate sebum production and intracellular lipid droplet formation in sebocytes.^6^, ^25^, ^26^ Ozenoxacin (10–100 μmol/L) dose‐dependently suppressed insulin‐induced mRNA expression of *DGAT‐1*, *SCD‐1*, and *perilipin‐1*, with a maximal inhibition of 72.5%, 74.8%, and 79.2%, respectively, at 100 μmol/L in a dose‐dependent manner (Figure 3). Similarly, ozenoxacin exhibited similar suppression in 5α‐DHT‐treated cells, with maximum inhibitions of 169.2%, 107.8%, and 131.8% at 30 μmol/L for *DGAT‐1*, *SCD‐1*, and *perilipin‐1*, respectively (see Figure 4). Although nadifloxacin exhibited dose‐dependent inhibition of *DGAT‐1*, *SCD‐1*, and *perilipin‐*1 mRNA expression in both insulin‐ and 5α‐DHT‐treated hamster sebocytes, its inhibitory effect was weaker compared with that of ozenoxacin (Figures 3 and 4). Furthermore, clindamycin showed negligible effects on *DGAT‐1*, *SCD‐1*, and *perilipin‐1* mRNA expression in both differentiated cells. Taken together, the results indicate that ozenoxacin may inhibit sebocyte lipogenesis by potentially suppressing the gene expression of *DGAT‐1*, *SCD‐1*, and *perilipin‐1* in insulin‐ and 5α‐DHT‐differentiated hamster sebocytes.

### 3.4 Inhibition of mTORC1 signaling by ozenoxacin in insulin‐ or 5α‐DHT‐differentiated hamster sebocytes

**FIGURE 5:**
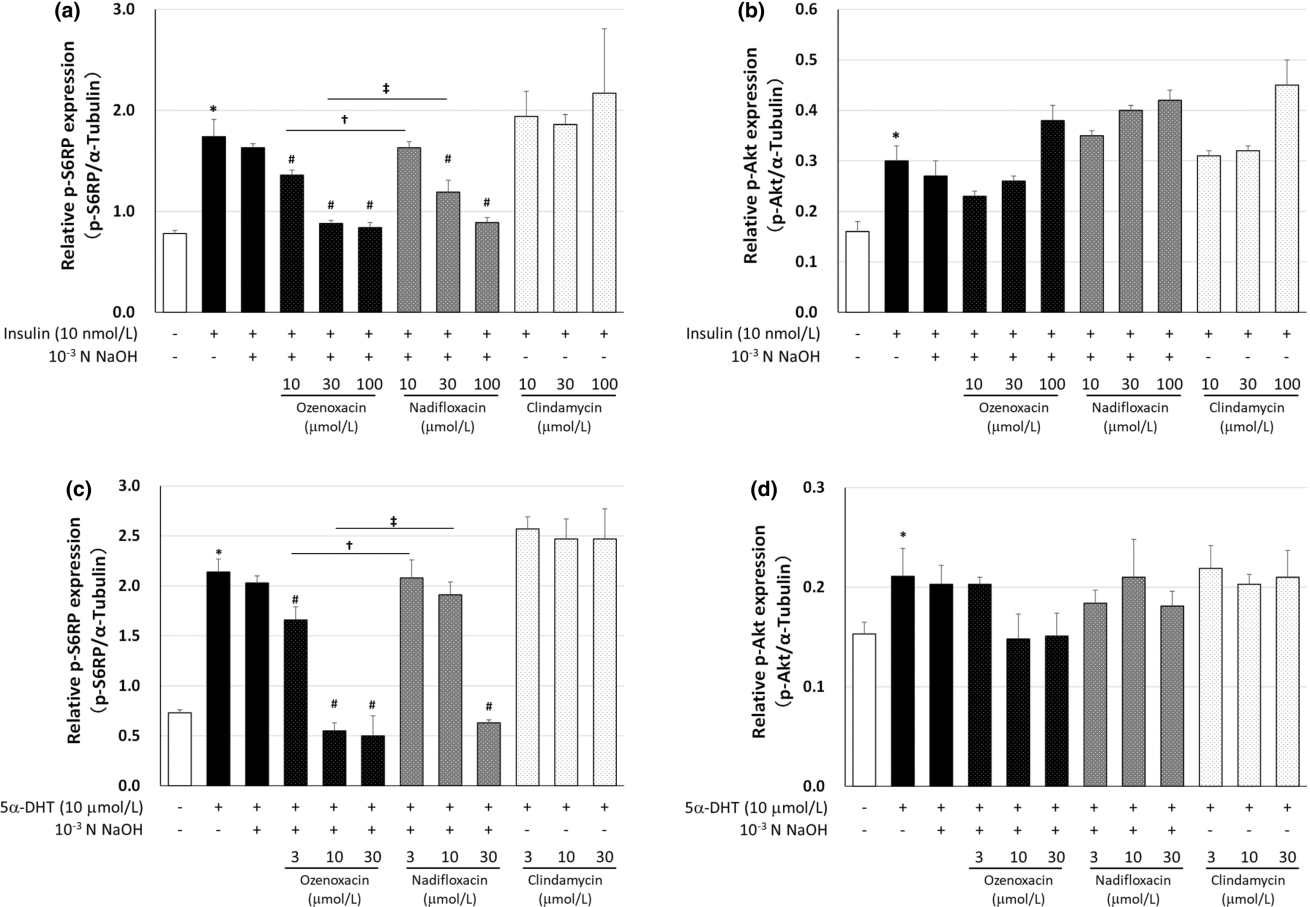
Effect of antimicrobial agents on phosphorylation of S6RP and Akt in differentiated hamster sebocytes. Hamster sebocytes were treated with ozenoxacin, nadifloxacin, and clindamycin in the presence or absence of insulin (a, b) and 5α‐dihydrotestosterone (5α‐DHT) (c, d), as shown in Figures 1 and 2, respectively. Cell lysates were prepared and intracellular p‐S6RP, p‐Akt, and α‐tubulin levels were measured as described in the Materials and Methods. Data are presented as means ± standard deviation of quadruplicate wells. *P < 0.05 compared with the non‐treated group (Student or Aspin‐Welch's *t*‐test). ^#^ *P* < 0.05 compared with insulin +10^−3^ N NaOH, or 5α‐DHT + 10^−3^ N NaOH (Tukey's multiple comparison test). ^†^ *P* < 0.05 compared with insulin + ozenoxacin 10 μmol/L, or 5α‐DHT + ozenoxacin 3 μmol/L (Tukey's multiple comparison test). ^‡^ *P* < 0.05 compared with insulin + ozenoxacin 100 μmol/L, or 5α‐DHT + ozenoxacin 10 μmol/L (Tukey's multiple comparison test).

Transcription factors, such as sterol regulatory element‐binding protein (SREBP)‐1 and peroxisome proliferator‐activated receptor, regulate the expression of *DGAT‐1*, *perilipin*, and *SCD‐1*.^27^, ^28^, ^29^ In addition, mechanistic/mammalian target of rapamycin complex 1 (mTORC1) modulates the activity of these transcription factors.^30^, ^31^ To elucidate the underlying mechanisms of the inhibition of sebocyte lipogenesis by ozenoxacin, we determined whether the mTORC1 signaling pathway was affected by ozenoxacin in insulin‐ and 5α‐DHT‐differentiated hamster sebocytes, compared with nadifloxacin and clindamycin. The phosphorylation of 40S S6RP and Akt, downstream and upstream of mTORC1, respectively,^32^ was attenuated (Figure 5). The levels of phosphorylated S6RO, increased by insulin‐ and 5α‐DHT, were reduced by ozenoxacin (maximally 89.6% at 100 μmol/L and 117.7% inhibition at 30 μmol/L, respectively) in a dose‐dependent manner (see Figure 5a,c). In contrast, the level of phosphorylated Akt remained largely unchanged (Figure 5b,d). Moreover, nadifloxacin inhibited the phosphorylation of S6RP in insulin‐ and 5α‐DHT‐treated sebocytes (88.3% inhibition at 100 μmol/L and 107.4% inhibition at 30 μmol/L, respectively), with minimal impact on phosphorylated Akt levels. Conversely, clindamycin showed little effect on phosphorylated S6RP and Akt levels in insulin‐ and 5α‐DHT‐differentiated hamster sebocytes. These results indicate that ozenoxacin, like nadifloxacin, preferentially inhibits the phosphorylation of S6RP in the mTORC1 signaling pathway in insulin‐ and 5α‐DHT‐differentiated hamster sebocytes.

### 3.5 Topical administration of ozenoxacin decreases skin surface TG levels in hamsters

**FIGURE 6:**
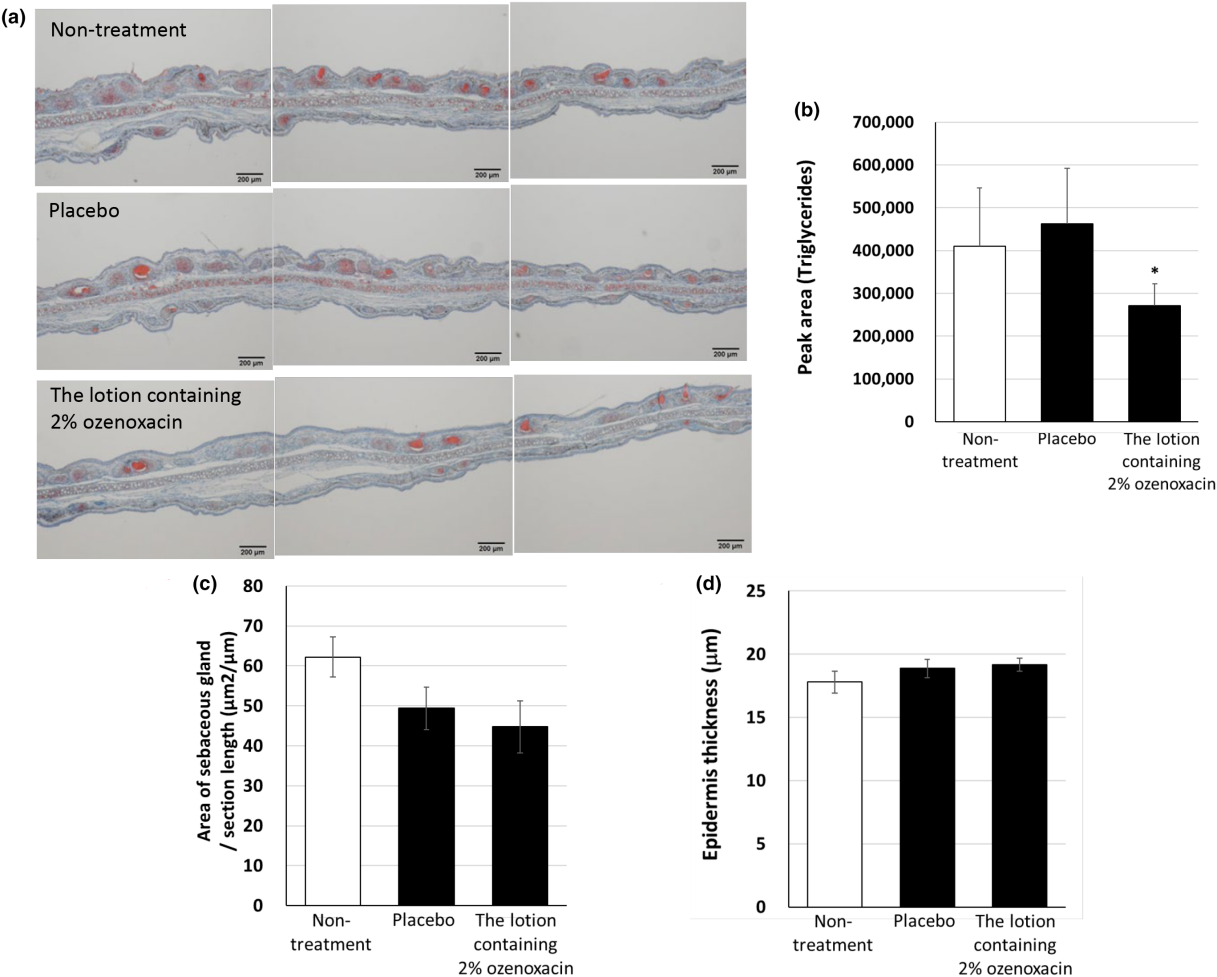
Decrease in triacylglycerol (TG) levels on the auricle skin surface of hamsters treated topically with a 2% ozenoxacin lotion. The lotion containing 2% ozenoxacin or a placebo was topically applied to both sides of the right auricle skin of hamsters once a day for 14 days. (a) The auricle tissues were fixed and stained with Oil Red O on day 15. Image analysis software evaluated the ratio of the total area of sebaceous glands to section length (b) and epidermis thickness (c) from three individual evaluation fields per tissue. (d) The right auricle skin was wiped with an acetone‐impregnated cotton on day 15. One hour after wiping, sebum on the skin surface was extracted three times with 3 mL of acetone for 1 min using glass test tubes. An automatic thin‐layer chromatography, Iatroscan, was used to analyze TG levels in the sebum from the skin surface of the hamster auricles. Data are presented as means ± standard deviation of tissues from 5 to 6 animals. *P < 0.05 compared with placebo (Fisher's least significant difference test).

We determined whether the topical administration of ozenoxacin decreased sebum production in vivo. After applying a lotion containing 2% ozenoxacin once daily for 2 weeks, Oil Red O staining revealed a weaker intensity of Oil red O positive sebaceous glands in ozenoxacin‐administrated auricle skin compared with the placebo group (Figure 6a). In addition, thin‐layer chromatography analysis revealed markedly reduced skin surface levels of TG in the ozenoxacin‐administrated auricles of hamsters (Figure 6b); however, no changes were observed in the area of the sebaceous gland or epidermal thickness between the ozenoxacin and placebo groups (Figure 6c,d), therefore the topical ozenoxacin administration effectively inhibited sebum production in hamsters.

## 4 DISCUSSION

Acne vulgaris, a prevalent skin condition primarily affecting adolescents, is associated with increased levels of androgens, such as testosterone and 5α‐DHT, which stimulate the proliferation of sebocytes and sebum production within the sebaceous glands.^3^, ^4^ Commonly used topical antibiotics, such as nadifloxacin and clindamycin, can treat inflammatory acne by impeding the proliferation of *C. acnes*.^10^, ^33^ Notably, Sato et al.^15^ reported that nadifloxacin and clindamycin also suppress sebum production in insulin‐differentiated hamster sebocytes. We found that ozenoxacin inhibits sebum production in 5α‐DHT‐differentiated hamster sebocytes in vitro. Furthermore, similar to ozenoxacin, nadifloxacin and, to a lesser degree, clindamycin inhibit sebum production in insulin‐treated and 5α‐DHT‐treated hamster sebocytes. These findings collectively indicate that ozenoxacin, nadifloxacin, and clindamycin exert inhibitory effects on sebum production in androgen‐differentiated sebocytes during adolescence.

Serum IGF‐1 levels are elevated in midpubertal individuals, correlating with increased sebum secretion.^3^ Furthermore, both IGF‐1 and insulin have been noted to directly affect the functions of sebaceous glands^34^ and elevate 5α‐reductase activity, leading to subsequent activation of androgen signaling by 5α‐DHT.^35^, ^36^ In our investigation, ozenoxacin inhibited insulin‐stimulated sebum production in hamster sebocytes. Consequently, these findings indicate that ozenoxacin and other topical antimicrobials, such as nadifloxacin and clindamycinantiacne, exhibit antiacne properties not only through their antimicrobial effects against *C. acnes* but also by suppressing sebaceous lipogenesis in sebocytes influenced by androgen, insulin, and IGF‐I, which are implicated in adolescent acne.

The differentiation of sebocytes exhibits characteristic features, including (i) augmented expression of *SCD‐1* and *DGAT‐1*, closely associated with sebaceous lipogenesis,^6^, ^25^ and (ii) intracellular accumulation of sebum as lipid droplets^26^ in humans and hamsters. SCD‐1 has been identified as the major isoform responsible for producing monounsaturated fatty acids in sebum,^25^ whereas DGAT‐1 is a rate‐limiting enzyme in TG biosynthesis.^27^ Akimoto et al.^26^ reported the involvement of perilipin‐1 in lipid droplet formation in differentiated hamster sebocytes. This study demonstrates that ozenoxacin suppressed the insulin‐ and 5α‐DHT‐induced expression of the *SCD‐1* and *DGAT‐1* genes in hamster sebocytes. In addition, ozenoxacin inhibited the formation of lipid droplets stimulated by both insulin and 5α‐DHT, concomitant with decreased perilipin‐1 expression. These findings suggest that the suppression of sebum production and accumulation by ozenoxacin could be associated with the downregulation of *SCD‐1*, *DGAT‐1*, and *perilipin‐1* in differentiated hamster sebocytes.

The activation of mTOR was reported to stimulate lipogenesis in response to various stimuli, including growth factors, insulin, nutrients, and oxygen.^37^ mTOR forms protein complexes, classified as mTOR complex 1 (mTORC1) and mTOR complex 2 (mTORC2), each exerting distinct functions. Specifically, mTORC1 is activated by insulin and IGF‐1, and promotes cell proliferation, differentiation, and lipid synthesis through the PI3K/Akt signaling pathway.^37^ In the present study, insulin increased the phosphorylation of S6K1, a downstream component of mTORC1, and Akt in hamster sebocytes. Furthermore, increased levels of phosphorylated S6K1 were no longer detectable on treatment with ozenoxacin (100 μmol/L), whereas there was a slight tendency toward an increase in phosphorylated Akt. Conversely, White et al.^38^ reported that low concentrations of testosterone (5 or 50 nM) activated mTORC1 in an Akt‐independent manner in mouse C2C12 myotubes. In contrast, high testosterone concentrations (500 nM) facilitated mTORC1 activity in an Akt‐dependent manner. This study demonstrated for the first time that 5α‐DHT increased the phosphorylation of S6RP and Akt in hamster sebocytes. Ozenoxacin inhibited the phosphorylation of S6RP, but not Akt, in 5α‐DHT‐treated sebocytes. When considered with our findings that both insulin‐ and 5α‐DHT‐induced sebum production were inhibited by the mTOR inhibitor KU‐0063794 and the PI3K inhibitor LY294002 (Supporting Information Figure S2), the results indicate that ozenoxacin selectively inhibits the activity of mTORC1 to decrease sebaceous lipogenesis in differentiated sebocytes.

Ozenoxacin, a quinolone antibacterial, features a unique structure, in which the fluorine atom at position six of the quinoline ring, a characteristic of new quinolone antibacterial drugs, is replaced by a hydrogen atom.^16^ Because of the structural disparity between nadifloxacin, another new quinolone antimicrobial, and ozenoxacin, we demonstrated that both agents reduce sebum production and accumulation, concurrently suppressing the expression of *SCD‐1*, *DGAT‐1*, and *perilipin‐1*, through the inhibition of mTORC1 activity in differentiated sebocytes. Yu et al.^39^ reported that levofloxacin, another quinolone antibiotic, inhibits the PI3K/Akt/mTOR pathway in MDA‐MB‐231 and MCF‐7 breast cancer cell lines. Although the effects of levofloxacin on sebum production have not been confirmed, quinolones or new quinolones containing quinoline rings, such as ozenoxacin and nadifloxacin, may regulate sebaceous gland function through mTOR signaling inhibition. Conversely, Eda et al.^40^ reported that clindamycin inhibits mTORC1 activation in human glioblastoma NGT41 cells. Our findings indicate that clindamycin slightly decreases sebum production and accumulation, exerting minimal effects on the expression of sebaceous lipogenesis‐related molecules or the phosphorylation of S6RP or Akt in differentiated sebocytes. Thus, the regulation of mTORC1 activity by clindamycin may vary among cell types.

Studies have indicated that mTORC1 plays a role in the pathogenesis of acne and upregulates the expression observed at lesion sites in acne patients.^41^, ^42^ Furthermore, the inhibition of the mTORC1 pathway suppresses insulin‐stimulated cell proliferation and lipid synthesis in human sebocytes,^43^, ^44^ which is consistent with our findings indicating that inhibiting mTOR activity reduces sebum production in insulin‐ and 5α‐DHT‐differentiated hamster sebocytes. Considering that hamster sebaceous glands share morphological and functional similarities with human sebaceous glands,^45^, ^46^ the suppression of sebum production by inhibiting mTORC1 activity may contribute to the therapeutic effect of ozenoxacin on acne.

In this study, the concentration of ozenoxacin in the in vitro experiments was higher compared with those required for antibacterial activity against *C. acnes* (Minimum Inhibitory Concentration (MIC) range ≤0.06 to 0.5 μg/mL).^22^ However, a clinical study using a 2% ozenoxacin lotion for acne vulgaris revealed that the mean concentration of ozenoxacin in the acne lesions in patients on day 3 of treatment was 53.6 μg/g.^22^ Our in vivo experiments yielded similar results using a lotion containing 2% ozenoxacin, wherein sebum on the skin surface decreased. At the same time, there was no change in epidermal thickness or sebaceous gland area in auricular skin tissues, therefore the decrease in the amount of sebum on the skin surface is likely the result of inhibiting sebum production by ozenoxacin‐mediated inhibition of mTORC1 activity in hamster sebaceous glands.

In summary, our findings provide novel evidence that ozenoxacin effectively inhibits sebum production and accumulation by suppressing the expression of *DGAT‐1*, *SCD‐1*, and *perilipin‐1* by inhibiting the mTORC1 signaling pathway in differentiated sebocytes, therefore ozenoxacin is a promising treatment for acne therapy by inhibiting microbial proliferation and effectively suppressing sebum production.

## CONFLICT OF INTEREST STATEMENT

Takamichi Kitano, Koki Fujikawa, Sachi Mori, and Tatsumi Matsumoto are employees of Maruho Co., Ltd. Toshikazu Koiwai and Takashi Sato are collaborators of Maruho Co., Ltd.

## Supporting information


Figures S1–S2.
