# Supplementary figures and images for: Ozenoxacin suppresses sebum production by inhibiting mTORC1 activation in differentiated hamster sebocytes
Source: J Dermatol. 2024 Aug 1;51(9):1187–98. doi: 10.1111/1346-8138.17409 (PMC11483923; doi:10.1111/1346-8138.17409)

Figure.S1

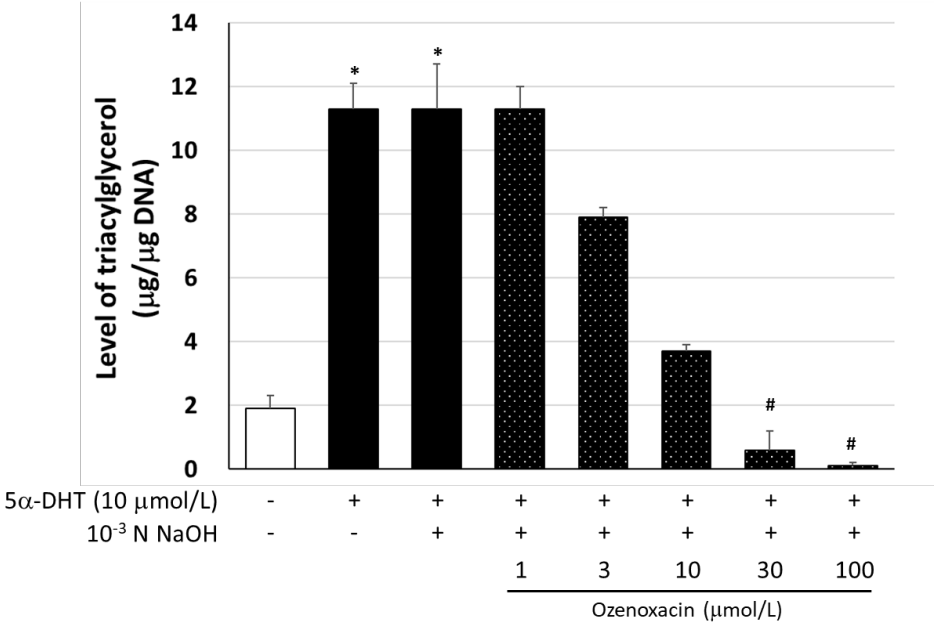

Figure.S2

[A]

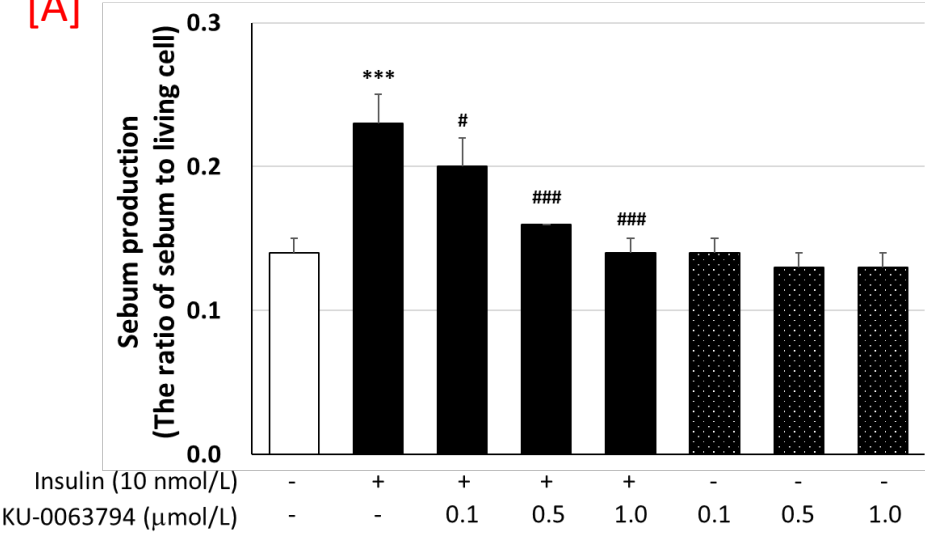

[B]

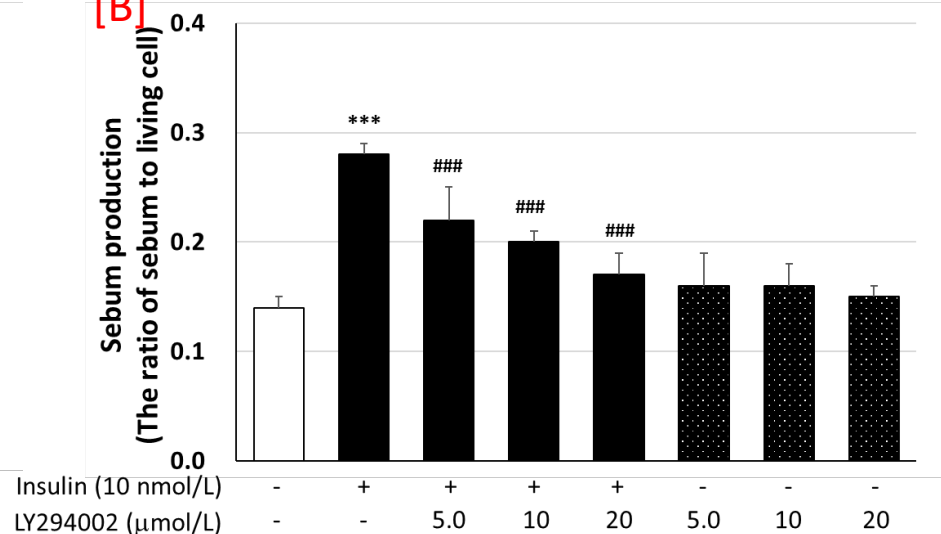

[C]

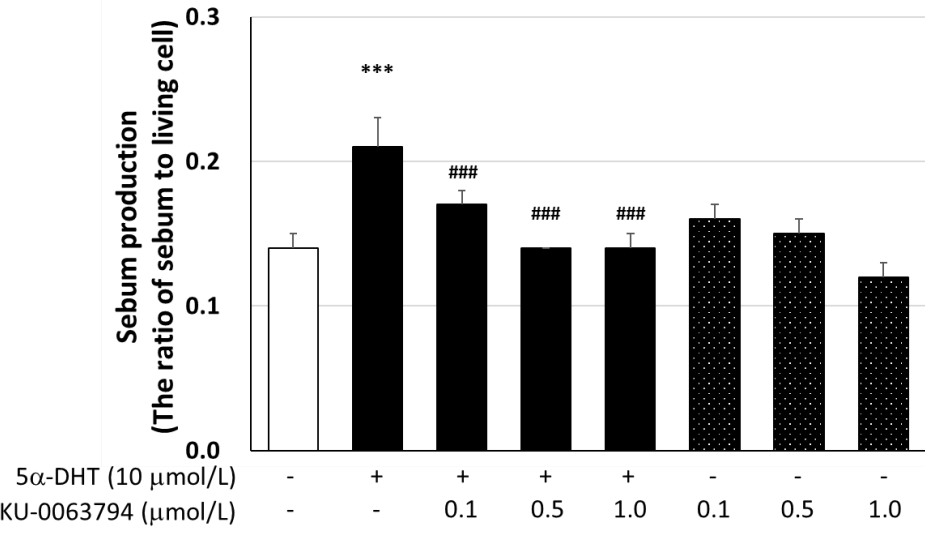

[D]

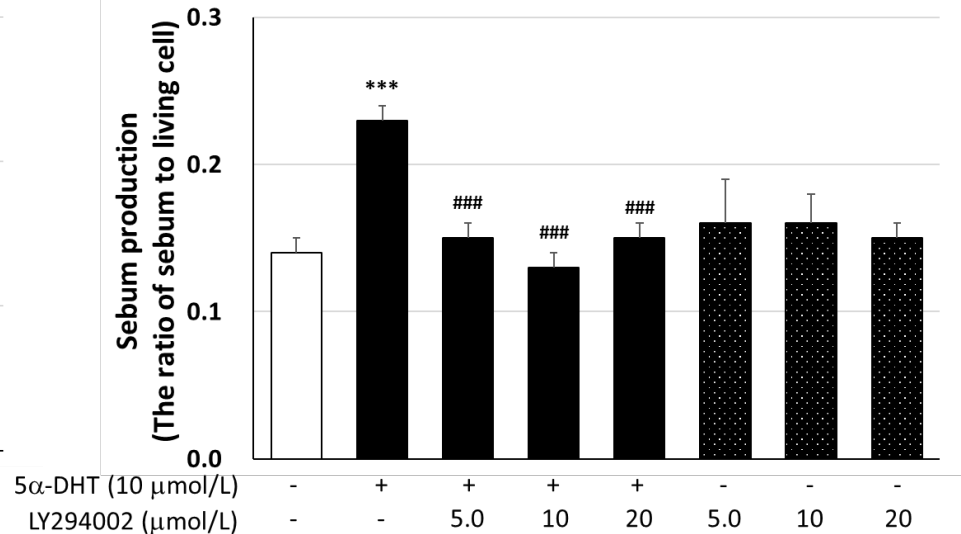

Supplement: Supplementary file 1 — Figures S1–S2. [file JDE-51--s001.pdf]
